# Exploring the psychological burden in a pancreatic cancer surveillance programme based on high-risk individuals: a Swedish cross-sectional study

**DOI:** 10.1136/bmjopen-2024-097814

**Published:** 2025-04-30

**Authors:** Anna Vesterberg, Ebba Asplund, Giulia Marras, Miroslav Vujasinovic, Cecilia Haddad Ringborg, Yvonne Wengström, Matthias Löhr

**Affiliations:** 1 Division of Surgery and Oncology, Department of Clinical Science, Intervention and Technology (CLINTEC), Karolinska Institutet, Stockholm, Sweden; 2 Department of Medicine Huddinge, Karolinska Institutet, Stockholm, Sweden; 3 Department of Upper Abdominal Diseases, Karolinska University Hospital, Stockholm, Sweden; 4 Division of Nursing, Department of Neurobiology, Care Sciences and Society, Karolinska Institutet, Stockholm, Sweden; 5 Karolinska Comprehensive Cancer Center, Stockholm, Sweden

**Keywords:** Pancreatic disease, Mass Screening, Cancer genetics

## Abstract

**Abstract:**

**Introduction:**

Pancreatic cancer, an aggressive cancer that presents with few or unspecific symptoms, has a poor prognosis. Thus, diagnosis at an early stage is vital for survival and a chance for curative treatment. Therefore, surveillance programmes for high-risk individuals are of the utmost importance. However, data on the psychological burden among participants in these programmes are limited.

**Aims:**

This study aimed to investigate the psychological burden for participants in a pancreatic cancer surveillance programme and explore whether the psychological burden was related to the individual’s risk level for pancreatic cancer.

**Methods:**

This single-centre cross-sectional study investigated cancer worry, anxiety, coping and perceived physical and mental health using a digital questionnaire, including the following instruments: Cancer Worry Scale (CWS), State–Trait Anxiety Inventory (STAI), 13-Item Sense of Coherence and 12-Item Short-Form Survey. The invited participants (n=413) were healthy individuals with an increased risk of pancreatic cancer enrolled in a pancreatic cancer surveillance programme.

**Results:**

The results indicated high cancer worry among respondents (n=78) with high scores on CWS (mean, 16.45). The majority (69.3%) had scores indicating high cancer worry (*≥*14). Anxiety was not equally high among respondents (mean STAI-T, 35.13; STAI-S, 35.9). Female sex and younger age were significantly correlated with higher cancer worry and anxiety (p < 0.001). Outcomes in coping and perceived health were similar to those of the normal population.

**Conclusions:**

Cancer worry is particularly high among participants. No correlation was found between the risk level and psychological burden.

**STRENGTHS AND LIMITATIONS OF THIS STUDY**

- A high response rate (67%) among the invited participants strengthens the reliability of the findings and reduces the risk of response bias.
- The study utilised validated and widely used psychological assessment instruments (12-Item Short-Form Survey, 13-Item Sense of Coherence, State–Trait Anxiety Inventory and Cancer Worry Scale), ensuring reliable and comparable results.
- The use of digital questionnaires minimised data entry errors and ensured standardised data collection.
- The sample size (n=78) was relatively small, limiting subgroup analyses, such as those based on specific genetic mutations.
- The study did not include data on participants’ personal or family history of cancers other than pancreatic cancer, which may have influenced their psychological burden.

## Introduction

 Pancreatic ductal adenocarcinoma (PDAC) is the deadliest cancer worldwide. Eighty percent of patients were in incurable stages at the time of diagnosis. Of those who undergo surgery, 80% will present with local or distant metastasis. This specific cancer has a low 5-year survival rate of <10%.^1^

The only curative treatment for PDAC is surgical resection; however, only pre-cancerous or early-stage PDAC is resectable and hence curable. Early-stage PDAC is usually clinically silent; therefore, methods of early detection are needed to detect the disease at an earlier stage. Screening programmes are therefore crucial.^2^ The lifetime risk of developing pancreatic cancer is 1.5% or 1 in 67.^3^

The low incidence of pancreatic cancer makes population-based screening non-feasible.^4^ However, screening is recommended for high-risk individuals (HRIs) with a higher lifetime risk of developing PDAC.^5^ MRI and/or endoscopic ultrasonography with at least 12-month intervals is recommended. Family history, age and germline mutation status are factors that determine eligibility for screening.^6^ HRIs are generally considered to have a 5% lifetime risk or a five-fold relative risk of developing PDAC. HRIs can be categorised into two groups: those with familial pancreatic cancer (FPC) and those with hereditary cancer syndromes, each with distinct population prevalence and associated pancreatic cancer risk.^3^

For individuals with familial pancreatic cancer, screening is recommended depending on how many and which family members have received a diagnosis of PDAC according to International Cancer of the Pancreas Screening (CAPS).^6^ In FPC, HRIs are defined as two or more first-degree relatives (FDRs) or one FDR and another relative on the same side of the family diagnosed with pancreatic cancer.^3 6^ The screening programme in Stockholm includes patients according to the CAPS criteria as standard clinical practice.^6^ Sometimes, individuals with only one affected FDR express worry about the risk of PDAC.^7 8^ In response, some programmes – including the screening programme in Stockholm – have started offering surveillance to individuals who do not meet the HRI requirement but who express worry.

Surveillance is recommended for individuals with germline mutations in genes associated with hereditary cancer syndromes, including *BRCA2*, *ATM*, *PALB2*, *CDKN2A*, *STK11*, *MLH1* and *MSH2*. Recommendations vary by gene and are outlined in current guidelines.^6^

Data on the psychological impact related to screening for PDAC are limited.^9^,^11^ Within screening programmes in the general population, anxiety among participants appeared to be low according to a systematic review on psychological distress associated with cancer screening.^12^ Moreover, the prognosis of the diagnosis screened for may affect the level of distress and worry. For pancreatic cancer, with its poor prognosis, the psychological burden among participants might be different.

In a previous study on worry, anxiety and depression for HRIs in the Netherlands using the Cancer Worry Scale (CWS), individuals who participated in intensified screening for PDAC were associated with a higher level of cancer worry than those who attended screening at a regular level (annual screening). Overall anxiety and depression did not change during intensified screening or after, as measured using the Hospital Anxiety and Depression Scale (HADS).^13^

More knowledge about the effects of screening on worry and anxiety is needed to evaluate the current screening methods. The experience of participating individuals is an important aspect and could yield valuable information. More insight into the topic could help improve programmes and suggest ways to better support these individuals. Therefore, this study aimed to investigate whether individuals with a higher risk of pancreatic cancer have a higher psychological burden than those who do not when participating in a screening programme.

## Materials and methods

### Study design

A cross-sectional assessment of the effect on individuals included in the pancreatic cancer screening programme at Karolinska University Hospital, Stockholm, Sweden, was conducted.

### Patient and public involvement

The design of the study and the inclusion of questionnaires had input from the patient organisation Pancreatic Cancer Europe and Swedish patient organisation Palema.

### Inclusion and exclusion criteria

The participants were individuals who took part a screening programme for high-risk PDAC, with so far no suspicion of an active tumour disease. Besides having chronic or autoimmune pancreatitis and pancreatic cystic neoplasms, such as intraductal papillary mucinous neoplasms (IPMNs), they were healthy individuals. The participants had been in contact with the pancreatic cancer surveillance programme, either for follow-up in the Stockholm region or for consultation while having follow-up in their home region. They were contacted via the quality register if they were free of pancreatic cancer or had been identified by the physician treating them.

The participants were divided into two subgroups based on whether they met the criteria for HRIs eligible for surveillance according to CAPS 2020 management recommendations. The requirement for recommendations was 80% unanimity.^6 12^ These two subgroups are referred to as ‘stringent HRIs’, fulfilling the CAPS criteria, and ‘non-stringent HRI’, who did not fully meet the CAPS criteria but were still included in surveillance in Stockholm due to an estimated increased risk.

A stringent HRI was defined as follows:

- All patients with Peutz–Jeghers syndrome (carriers of a germline *LKB1/STK11* gene mutation).
- Carriers of a germline *CDKN2A*, *BRCA2*, *PALB2*, *ATM*, *MLH1*, *MSH2* or *MSH6* gene mutation with at least one affected FDR with pancreatic cancer.
- Individuals with at least two affected relatives on the same side of the family, of whom at least one is an FDR to the individual considered for surveillance.^6^

A non-stringent HRI was defined as individuals with similar traits and considered to have some increased risk of pancreatic cancer but who did not fully meet the above criteria.

The participants either had relatives diagnosed with PDAC or had a gene mutation that increases the risk of PDAC, or both. In addition, they could also have the phenotype IPMN (cystic lesions). Patients with branch duct IPMN were included, whereas patients with indications for surgery and not surveillance, such as main duct IPMN, high-risk stigmata or other malignancy indicators, were excluded.

Depending on the specifics and the combinations, the risk varies and determines whether or not an individual was classified as a stringent HRI. For details on the two subgroups in this study, see table 1.

**Table 1:** Characteristics of the stringent and non-stringent populations

Stringent and non-stringent study populations								
		Totaln=278		Non-stringentn=88		Stringentn=190		P value
Cystic lesion	No cysts	107	39.1%	34	40.0%	73	38.6%	
	Cysts	167	60.9%	51	60.0%	116	61.4%	0.829
Sex	Female	188	67.6%	64	72.7%	124	65.3%	
	Male	90	32.4%	24	27.3%	66	34.7%	0.216
Genetic mutation	No mut.	192	69.1%	43	48.9%	149	78.4%	
	Mutation	86	30.9%	45	51.1%	41	21.6%	< 0.001
AgeFollow-up time	(*mean*)	59	SD 11	56	SD 11	61	SD 11	< 0.001
	(*months*)	83	SD 53	71	SD 46	88	SD 56	< 0.05

mut, mutation.

### Data collection

#### Data sources

Participant data were extracted from a quality control database at Karolinska University Hospital in Huddinge. Updated information about the participants was retrieved through electronic medical records to ensure that the participants continued to meet the inclusion criteria.

#### Questionnaires

Questionnaires were used to assess perceived health, coping, anxiety and cancer-related worry. The questionnaire included the following instruments: 12-Item Short-Form Survey (SF-12), 13-Item Sense of Coherence (SOC-13), State–Trait Anxiety Inventory (STAI) and Cancer Worry Scale (CWS). The research group that conducted the study chose the questionnaires based on experience.

Information and instructions were sent by letter to the participants on 25 October 2023, including a QR code to an electronic questionnaire that was accessed via mobile phone, tablet or computer. All data entries and modifications were tracked and connected to personal log-in credentials. Consent was collected electronically before the start of the survey. The survey closed on 14 November 2023.

SF-12 was used to measure perceived health. This instrument was originally developed in the USA for use in large-scale health measurement and monitoring efforts, in which the focus was on overall physical and mental health outcomes. It contains 12 items and is a shorter alternative to the original 36-Item Short-Form Health Survey (SF-36).^14^ High scores represent good physical and mental health. The test score is norm-based standardised, where a score of 50 and an SD of 10 is achieved in the general US population. The test yields two total scores: the physical component summary (PCS) score and mental component summary score (MCS).^15^

The SOC-13 was used to assess stress and coping. Sense of coherence is explained as a characteristic trait that makes the individual more resilient to stressors in daily life and more capable of staying well and healthy. It consists of 13 items, and responses are given on a 7-point scale. The total score ranges from 13 (low sense of coherence) to 91 (high sense of coherence).^16 17^

The STAI assesses dispositional (trait) and transitory (state) types of anxiety. State anxiety can be described as an unpleasant emotional state or condition, and trait anxiety is a relatively stable personality trait. Anxiety incorporates feelings of tension, nervousness and worry. The instrument is a self-report inventory consisting of two questionnaires, STAI-T (trait) and STAI-S (state), each consisting of 20 items.^18^ The responses are given on a 4-point scale. A total score is calculated by adding the score from each question, with some of the questions’ scores being reversed. A high score indicates high anxiety.^19^

The CWS was used to measure cancer worry. This instrument consists of eight items, to which responses are given on a 4-point Likert scale. The total score ranges from 8 to 32, with a higher total score indicating greater worry about cancer and/or greater effect on mood and daily functioning because of cancer worry.^20^

### Data analysis

To analyse the responses from SF-12, the manual for SF-12 was used.^15^ Question numbers 1, 8, 9 and 10 have reverse scoring. SF-12 total scores are calculated through a four-step process that entails reverse scores, creating indicator variables from the questions, weighting these variables and adding a constant so the scores are standardised to have the same mean as SF-36. For this, a syntax code for SPSS was used. The responses from the SOC-13 questionnaire are presented as a score between 1 and 7 for each of the 13 questions. Question numbers 1, 2, 3, 7 and 10 have reverse scoring and were reversed before calculating the total score. After the total score was calculated as no to, they were categorised as low, moderate and high scores. The higher the score, the better the sense of coherence and the better the ability to deal with stressors. Low scores were defined as scores between 30 and 60, moderate scores as between 61 and 75, and high scores as between 76 and 91. Similar categorisation is found in other studies.^21^,^24^ The responses on STAI yielded a score from 1 to 4 for each of the 20 questions, which together gave the total score. There were two total scores, STAI-T and STAI-S, with 20 questions each. Some of the questions have reverse scoring. The total score is presented with descriptive analysis. The scores were categorised as moderate to high anxiety, with a cut-off of 40 or more. Scores of 39 and lower were categorised as having none to experiencing minimal symptoms of anxiety. Cut-off values were selected according to earlier studies.^25 26^ The responses to the CWS were calculated by adding the scores from each of the eight questions to give a total score ranging from 8 to 32. The scores were then categorised as high and low scores, representing high and low cancer worry, respectively. The cut-off values used were 13 or below representing low cancer worry and 14 and above representing high cancer worry. The same cut-off values have been used in earlier studies.^20 27^ Patients with missing data were excluded from the analysis of the questionnaire with missing data.

### Statistics

Parametric methods were used for normally distributed data and non-parametric methods for non-normally distributed data. Pearson’s χ^2^ tests were used to describe and compare participant characteristics.^28^ Student’s t-test or ANOVA was used, as appropriate, to detect significant differences between groups for continuous variables.^29^ IBM SPSS Statistics for Mac (version 28.0.1.1, IBM Corp., Armonk, NY, USA) was used for data analysis.

### Ethical considerations

The study was approved by the local institutional review board of Swedish Etikprövningsmyndigheten (EPN DNr. 2023–0573801).

## Results

Of 413 eligible participants, 278 responded to the questionnaire (see online supplemental figure S1) and 248 completed the questionnaire. Of these participants, 190 were stringent HRIs and 88 were non-stringent HRIs. The mean age for this study population was 59 years, and the mean time during which they had been under screening follow-up was approximately 7 years.

Within the stringent and non-stringent subgroups, characteristics were fairly similar, except for the presence of a genetic mutation, where a larger group within the stringent subgroup did not have a known gene mutation. Non-stringent HRIs were also younger and had shorter follow-up times (see table 1).

### Perceived health: SF-12

The mean PCS and MCS scores from the SF-12 questionnaire were 50.7 and 51.8, respectively, for the whole population. Comparing results within subgroups in table 2, there was a difference in MCS scores between female and male participants (p<0.05).

**Table 2:** Results from SF-12 physical and mental scales

		Mean	MCS-12				
			Median	SD	Mean	Median	SD
Total		50.68	54.07	8.39	51.82	55.34	9.37
Sex	Female	50.25	54.03	8.64	50.89*	53.89	9.26
	Male	51.57	54.22	7.82	53.72*	56.98	9.38
Stringent	Stringent	50.81	54.19	8.36	52.16	55.93	9.55
	Non-stringent	50.40	53.96	8.49	51.04	53.49	8.98
Cystic lesion	Cyst	50.36	53.80	8.23	51.95	55.68	9.65
	No cyst	50.94	54.19	8.68	51.80	55.18	9.03
Presence of gene mut.	Gene mutation	50.25	54.78	9.34	52.05	54.49	8.30
	No known mutation	50.88	53.80	7.93	51.71	55.68	9.86

Valid n=236.

*p<0.05.

mut, mutation.

### Coping: 13-Item Sense of Coherence

The mean score for coping was 69.9 on a scale from 13 to 91 (with higher scores indicating a higher sense of coherence). Men tended to score high to a greater extent, as seen in table 3. Those who had a known gene mutation scored moderate to high more often, but the correlation was not significant. However, the analysis showed that male sex and older age were significantly correlated to higher scores (p<0.05). No statistical significance was found between the stringent and non-stringent participants.

**Table 3:** Results for SOC-13 and categorised scores

SOC-13								
		Mean	Median	Range	SD	Low scores	Moderate scores	High scores
Total		69.88	72	54.00	11.56	23.8%	40.4%	35.7%
Sex	Female	68.61*	71	54.00	12.09	28.0%	39.1%	32.9%
	Male	72.65*	74	43.00	9.84	14.9%	43.2%	41.9%
Stringent	Stringent	70.34	73	54.00	12.16	23.8%	37.5%	38.8%
	Non-stringent	68.89	70	41.00	10.18	24.0%	46.7%	29.3%
Cystic lesion	Cyst	70.37	72	54.00	11.92	24.1%	38.0%	38.0%
	No cyst	69.35	72	52.00	11.12	23.2%	43.2%	33.7%
Presence of gene mut.	Gene mutation	70.18	71	43.00	10.58	17.1%	48.7%	34.2%
	No known mutation	69.74	73	54.00	12.03	27.3%	36.0%	36.6%
Age, mean		*				57.16	58.38	60.73

Valid n=235.

* Significant p<0.05.

Correlations between baseline characteristics and scores with x² analysis and linear regression

mut, mutation.

### Anxiety: State–Trait Anxiety Inventory

Anxiety showed a mean score of 35.13 for STAI-T and 35.91 for STAI-S. More than 30% had scores indicating moderate to severe anxiety, as shown in online supplemental table S1, 32.7% and 31.4% scored high for state and trait anxiety, respectively. There was a significant correlation between female sex and moderate to severe anxiety. The analysis shows a significant correlation with higher anxiety for female sex and younger age. The study also shows a tendency for less anxiety if the individual has a known genetic mutation, although these results were not significant.

### Worry about cancer: Cancer Worry Scale

When assessing cancer worry, the results showed a mean score of 16.45, indicating that close to 70% of participants have high cancer worry (see table 4).

**Table 4:** Results from the cancer worry scale

CWS							
		Mean	Median	Range	SD	Low cancer worry	High cancer worry
Total		16.45	16	22.00	4.83	30.7%	69.3%
Gender	Female	17.23*	17	22.00	4.82	24.1%*	75.9%*
	Male	14.59*	15	18.00	4.37	46.4%*	53.6%*
Stringent	Stringent	16.13	16	22.00	4.74	33.5%	66.5%
	Non-stringent	17.09	16	20.00	4.98	25.0%	75.0%
Cystic lesion	Cyst	16.39	16	22.00	4.57	31.1%	68.9%
	No cyst	16.58	16	21.00	5.27	30.1%	69.9%
Presence of gene mutation	Gene mutation	17.12	16	22.00	5.25	27.8%	72.2%
	No known mutation	16.14	16	19.00	4.61	32.1%	67.9%
Age, mean		**				60.75	58.43

*Significance level p<0.001, **Significance level p<0.05, Valid n=231.

Cancer worry was categorised into low (cut-off <14) and high (cut-off>14) cancer worry. Correlations between baseline characteristics and scores were analysed using the χ2 test and linear regression.

There was a significant correlation between high cancer worry and female sex, where 75.9% of the female participants had high cancer worry compared with 53.6% of men (p<0.001). The analysis showed significant correlations between sex and age, with being female (p<0.001) and having a younger age (p<0.05) yielding more worry. No significant difference was found between stringent and non-stringent participants. 

## Discussion

While it is the intention of surveillance to provide security for HRIs developing pancreatic cancer, even being part of the programme carries a risk of anxiety. Limited studies have investigated the psychological burden of participants in pancreatic cancer surveillance. In the current study, 278 surveillance participants responded to questionnaires assessing cancer worry, anxiety, perceived health and coping. The results indicate that the participants have high cancer worry. The cancer worry among younger female participants was significantly higher than among the other participants. In addition, younger patients and female participants also suffered from significantly more anxiety.

Despite the differing levels of pancreatic cancer risk between stringent and non-stringent HRIs, this study did not observe significant differences in psychological burden between these groups. This suggests that the awareness of increased risk, regardless of its magnitude, may have a uniform psychological effect on individuals under surveillance. These findings underscore the importance of providing comprehensive psychological support to all HRIs, tailored to their specific needs rather than solely their risk level.

Regarding quality of life, the participants scored close to the scores of the normative population presented in the scoring manual.^15^ The physical scores could be comparable to normative levels, since this study population generally consisted of healthy individuals. However, studies of different surveillance programmes present lower physical scores. For example, studies on individuals under skin and lung cancer surveillance reported drastically lower PCS scores.^30 31^

Compared with the normative population, scores regarding the sense of coherence within this surveillance group were considered high. Since only about 24% scored low, this study population seems to have the ability to handle challenges in life well. An important consideration is that different studies categorised high, moderate and low scores differently. There is no universal cut-off value for this questionnaire.^24^ If other cut-off values had been used for this study, the results from the categorised scores would have been different. However, the mean scores remain universal for all studies using SOC-13.

Using the STAI instrument, female and younger participants scored significantly higher on both state and trait anxiety scales. About a third of the study population had scores indicating moderate to severe symptoms (32.7% on STAI-S and 31.4% on STAI-T). These results are similar to those of the normative population.^19^ The anxiety levels in the surveillance participants seem to be comparable to those of the general population. By contrast, in a study in China including women participating in cervical cancer screening, the mean STAI-S score was 42.72 before the screening procedure, and 70.3% had scores indicating moderate to high anxiety (scores of 40 or higher) pre-procedure.^32^ High scores were also reported in a Japanese study investigating the psychological burden in individuals with different genetic conditions receiving genetic counselling.^33^ Conversely, lower scores were obtained in other surveillance programmes. A study with participants under ovarian cancer screening had mean STAI-S and STAI-T scores of 30 and 29, respectively.^34^ In a Danish study, women who had been referred to an obstetrician or a gynaecologist due to an abnormal cervical screening result also showed lower STAI-Sscores .^35^ Participants of a lung cancer screening programme also had low scores on three different occasions.^31^ Additionally, a Swedish study evaluating anxiety in colorectal cancer screening participants concluded that female sex was associated with higher anxiety.^36^ Patients in the colorectal study had a mean score of 34.1, which was similar to the scores yielded in this study on HRI. However, even if the means were similar, the categorised scores varied, with the majority of the HRIs having moderate to high symptoms. In a colorectal study, 21% of the participants scored high compared with 32.7% of the HRIs who had scores in the same category.^36^ Lastly, another relevant comparison of the population was with the patients under surveillance for IPMN in a Finnish study of health-related quality of life and anxiety levels. The STAI-S results showed that anxiety did not increase or decrease following surveillance participation.^23^ Altogether, when comparing the scores in the present study with those of other studies, anxiety seems to be at normative levels and does not seem to be affected by participation in PDAC surveillance or having a higher risk of PDAC. Mixed results were obtained within screening populations in other studies, with scores both higher and lower than the scores in the present study. The scores in the present study are similar to the reference normative population for the STAI instrument and to scores from a colorectal study in Sweden.^36^

The CWS showed a high degree of cancer worry among the participants in the present study. Being a woman and of younger age were correlated with more worry. A similar correlation was found in another study investigating cancer worry in the general population (not predisposed) in Sweden, where women also scored significantly higher than men.^20^ Participants in another pancreatic cancer surveillance programme in the Netherlands were asked to answer CWS on four different occasions during surveillance. The mean score for those occasions was 13.2, and 51%, 52%, 43% and 33% of the participants scored 14 or above on these occasions.^37^ Because of the poor prognosis of pancreatic cancer and the fact that many of these risk patients have family members who have suffered from this disease, high scores for cancer worry might be expected. As in the present study, other studies have also found a correlation with sex and age, where female and younger individuals tend to be more worried about cancer.^38^,^40^

To address the psychological burden of pancreatic cancer screening, it is essential to integrate targeted psychological support into these programmes. Providing access to psychologists or counsellors can offer patients a safe space to discuss their concerns, alleviating anxiety and improving overall well-being. By addressing the specific needs of vulnerable groups, screening programmes can become more patient-centred and effective, ensuring continued participation and a better patient experience.

### Strengths and weaknesses

The response rate in the present study was high, with 67% of all those who were invited participating (and 60% completing the questionnaire). The number of participants (n=278) was, however, limited due to the size of the surveillance group. Additionally, certain factors that could influence psychological burden were not accounted for in our analysis. Specifically, we did not collect data on personal or family history of cancers outside of PDAC, which may be relevant for understanding psychological distress. Furthermore, we did not analyse differences in psychological burden based on specific genetic mutations, which could influence perceived cancer risk and worry. While our sample size may not have been large enough to detect statistically significant differences for these variables, future research should explore their effect on larger cohorts. The study could be extended and conducted in other PDAC surveillance groups in other regions or countries to further strengthen the results.

Of the 278 participants, 32% were non-stringent HRIs and 68% were stringent HRIs. The female population was twice the size of the male population. Cystic lesions and the presence of a known gene mutation were also not equally distributed. These could be contributing factors to errors in the results. The study participants might also be more worried in general or have more anxiety. As a result, those who experience high anxiety levels might not participate in this study.

By using well-known and validated questionnaires, others can conduct similar studies in other populations. Moreover, the use of four different questionnaires gave the possibility to measure different aspects of psychological burden. It provided a broader insight into the psychological burden. Although all the questionnaires have been frequently used and validated, there might be a risk that the investigated aspect of psychological burden was not properly identified by each questionnaire. For instance, for the SF-12, one study raised a concern regarding the scoring algorithm used and expressed concerns about the risk of misleading and ambiguous results.^41^ Additionally, using these instruments as digital questionnaires limited the risk of handling errors when performing the analysis, which is considered a strength of the method.

### Conclusions

Cancer worry in participants undergoing PDAC surveillance is prominent, with nearly 70% of participants scoring high. When comparing these results with those of other studies for other cancer types, most studies do not present such high scores. Women and those of a younger age had significantly more cancer worry and anxiety. Anxiety levels for the total study population were close to normative levels. The knowledge gained from this study can be used to support these individuals better in screening programmes.

## Supplementary material

10.1136/bmjopen-2024-097814 online supplemental file 1

## Data Availability

Data are available upon reasonable request.
