# Supplementary material for: Exploring the psychological burden in a pancreatic cancer surveillance programme based on high-risk individuals: a Swedish cross-sectional study
Source: BMJ Open. 2025 Apr 30;15(4):e097814. doi: 10.1136/bmjopen-2024-097814 (PMC12049877; doi:10.1136/bmjopen-2024-097814)
Supplement: online supplemental file 1 [file bmjopen-15-4-s001.docx]

**Psychological burden in a pancreatic cancer surveillance program based on high-risk individuals**

**Supplementary Material**

**Figure S1:** A flow chart of the study population and the inclusion process.

**Table S1:** Results for STAI-T and STAI-S and categorized scores. Correlations between participant characteristics using Chi-square and linear regression.

| STAI | | | | | | | | | | | |
| --- | --- | --- | --- | --- | --- | --- | --- | --- | --- | --- | --- |
|  | | **STAI-S** | | | | | **STAI-T** | | | | |
|  | | **Mean (range)** | **Median** | **St. dev** | **No or minimal symptoms** | **Moderate to severe symptoms** | **Mean (range)** | **Median** | **St. dev** | **No or minimal symptoms** | **Moderate to severe symptoms** |
| Total | | 35.91 (49.00) | 33 | 11.05 | (150) 67.3% | (73) 32.7% | 35.13 (51.00) | 33 | 10.14 | (151) 68.6% | (69) 31.4% |
| Gender | Female | 37.49  * | 35 | 11.79 | (95) 61.7%* | (59) 38.3% * | 36.81  ** | 35 | 10.71 | (93) 62.4%* | (56) 37.6%* |
|  | Male | 32.39* | 30 | 8.25 | (55) 79.7%* | (14) 20.3%* | 31.62  ** | 30 | 7.80 | (58) 81.7%* | (13) 18.3%* |
| Stringent | Stringent | 35.21 | 32 | 11.30 | (105) 68.2% | (49) 31.8% | 35.14 | 33 | 10.60 | (101) 67.8% | (48) 32.2% |
|  | Non-stringent | 37.46 | 37 | 10.39 | (45) 65.2% | (24) 34.8% | 35.11 | 34 | 9.18 | (50) 70.4% | (21) 29.6% |
| Cystic lesion | Cyst | 34.77 | 32 | 10.83 | (89) 69.5% | (39) 30.5% | 34.81 | 34 | 9.89 | (87) 68.0% | (41) 32.0% |
|  | No cyst | 37.12 | 35 | 10.95 | (60) 65.2% | (32) 34.8% | 35.48 | 32 | 10.42 | (62) 69.7% | (27) 30.3% |
| Presence of  gene mut. | Gene mutation | 35.66 | 34 | 9.42 | (52) 70.3% | (22) 29.7% | 34.41 | 34 | 8.78 | (49) 74.2% | (17) 25.8% |
|  | No known mutation | 36.03 | 32 | 11.81 | (98) 65.8% | (51) 34.2% | 35.44 | 33 | 10.69 | (102) 66.2% | (52) 33.8% |
| Age mean |  | * |  |  | 60.19*** | 56.47*** | *** |  |  | 59.83 | 57.46 |

Scores ≥ 40 indicate moderate to high anxiety.
* Significant p < 0.01 . ** Significant p < 0.001. *** Significant p < 0.05.
mut. = mutation, St. dev = standard deviation.
