# Supplementary material for: Exploring the psychological burden in a pancreatic cancer surveillance programme based on high-risk individuals: a Swedish cross-sectional study
Source: BMJ Open. 2025 Apr 30;15(4):e097814. doi: 10.1136/bmjopen-2024-097814 (PMC12049877; doi:10.1136/bmjopen-2024-097814)
Supplement: Supplementary file 2 [file bmjopen-2024-097814.reviewer_comments.pdf]

PEER REVIEW HISTORY

BMJ Open publishes all reviews undertaken for accepted manuscripts. Reviewers are asked to complete a checklist review form and are provided with free text boxes to elaborate on their assessment. These free text comments are reproduced below.

ARTICLE DETAILS

Title (Provisional)

Exploring the psychological burden in a pancreatic cancer surveillance program based on high-risk individuals: A Swedish cross-sectional study

Authors

Vesterberg, Anna; Asplund, Ebba; Marras, Giulia; Vujasinovic, Miroslav; Haddad Ringborg, Cecilia; Wengström, Yvonne; Löhr, Matthias

---

VERSION 1 - REVIEW

**Reviewer**	**1**
**Name**	**Paiella, Salvatore**
**Affiliation**	**University and Hospital Trust of Verona**
**Date**	**20-Dec-2024**
**COI**	**None**

Thanks for assigning me to revise an interesting manuscript that deals with a topic I am keen on. The authors should be commended for the study and for having used the CWS in the context of pancreatic cancer surveillance, which is uncommon.

Please find below my considerations, comments, and concerns.

Minor:

1) The use of “individuals at risk”, since the title, between quotation marks is unusual, whereas high-risk individuals is appropriate. The authors may be willing to use this locution throughout the whole manuscript. They use both acronyms, IAR and HRI, whereas the most adopted is HRI, and, anyway, only one should be chosen, preferably HRI.

2) Phrasing is sometimes poor, e.g.:

- There are two groups of IAR: familial pancreatic cancer (FPC) and hereditary cancer syndromes, with varying population prevalence and pancreatic cancer risk.

- However, individuals with a higher risk of developing cancer than the general population might

experience this differently (they should generalize and stay focused on PDAC surveillance)

- “to support these individuals better…” To better support these individuals.

- “Individuals participating in a screening program for high-risk PDAC, with no signs of active tumor disease.”: Individuals are at risk, not PDAC. What does it mean “with no signs of active tumor disease”?

- “They were contacted via the quality register, if still alive”: of course, they were alive if they were contacted. Please rephrase or remove.

3) The paragraph starting with “Surveillance among” and closing with “pancreatitis attack” can be removed, and the reader will be addressed to current recommendations/guidelines. This will shorten the introduction, which is currently too long and would benefit from some cuts about general features of PDAC surveillance (modalities and inclusion criteria).

4) The authors state that “There is limited data on psychological impact related to screening for PC”, referring only to the manuscript of Cazacu et al. They are missing other manuscripts published afterward: PMIDs: 38201514, 32108397.

5) PDAC or PC?

6) The authors should state who selected the questionnaires to (self)administer. A psychologist? A psycho-oncologist?

7) The phrase “In response, some programs – including the screening program in Stockholm – have started offering surveillance to individuals who do not meet the IAR requirement, but who express worry”. What do the Authors mean? In a few countries, pancreatic cancer surveillance is considered standard clinical practice. In others, it is made within research programs, registered and IRB-approved. What is currently done in Sweden-Stockholm? The Authors may be willing to expand on this and state it clearly.

8) Patient enrollment for prospective psychological evaluation: it is unclear if the Authors want to underline a selection bias or describe how patient selection was made.

9) From “The participants were divided”.. to “Cystic lesions”. This part may benefit from a more concise and precise description of the risk categories of the HRI involved.

Main criticisms:

1) The main criticism is the inclusion of “the phenotype IPMN”, which per sé is sibylline. What is a phenotype IPMN? MD-IPMN? BD-IPMN? Any info about worrisome features/high-risk stigmata? Ca 199 rise? Which are the genetic mutations the Authors refer to?

Is this population merging the reason why they chose “individuals at risk”, underlying that a case-mixing was made including those having heredo-familial predisposition and cystic precursor lesions? The risk of malignant transformation is tremendously different regarding the various types of IPMN. The comparison of the psychological burden between this population and HRI with heredo-familial predisposition should be made prudently. Comparing the two main categories of HRI under surveillance sounds appropriate and

scientifically valid, as well as among the categories of IPMN. Others have already made these comparisons.

Any comparisons and speculations among such different populations may be weak or clinically unsuitable, and the message delivered is misleading.

2) The authors should correct their statistics for important variables that may ultimately impact the psychological assessment: a personal history of cancer (e.g., those HRI having genetic mutations may be more prone to have psychological distress), type of genetic mutation (HRD vs. Non-HRD), and family history of cancer (presumably rare in the cystic lesions group). Maybe the data are not large enough to achieve statistical significance, yet this deserves further exploration or at least mention among the weaknesses (which not mention at all the fact that "cystic lesions", in general, were included).

**Reviewer**	**2**
**Name**	**Drott, Jenny**
**Affiliation**	**Linköping University**
**Date**	**28-Dec-2024**
**COI**	**None**

The paper investigates an interesting topic about the psychological burden in a pancreatic cancer surveillance program. Your research presents a significant contribution to the field of psychological burden in a pancreatic cancer surveillance program, offering valuable insights and advancing our clinical understanding of this specific topic.

The thoroughness of your methodology and the clarity of your results are particularly impressive, making this study a crucial reference for future research. Your work not only addresses key questions but also opens new avenues for exploration and supportive cancer care, underscoring its importance and relevance in women and younger individuals with more cancer worry and anxiety in a pancreatic cancer surveillance program.

Comments.

1. Why were patients not involved in the design of this study? How might their involvement have impacted the study outcomes? Consider adding in limitations.

2. How were the criteria for high-risk PDAC determined, and what evidence supports these criteria?

3. Consider clarifying the specific criteria used to classify participants into stringent IAR and non-stringent IAR subgroups.

4. Consider clarifying how the risk of developing pancreatic cancer differ between the stringent and non-stringent groups, and the clinical implications of these differences.

5. How was the quality control database at Karolinska University Hospital maintained, and what measures were taken to ensure data accuracy?

6. What are the potential limitations of using electronic medical records to update participant information?

7. You mention that your response rate in your study was high, with 67% of all those who were invited participating but also indicates that 33% of the patients did not provide data, it is potentially response bias? limit the generalizability of your findings?

8. How were the questionnaires you used in the study validated for this specific population, and what are the potential limitations of using these instruments (in this specific population)?

9. What are the ethical considerations of contacting participants via the quality register, and how was consent managed?

10. How might your results impact future pancreatic cancer screening programs?

VERSION 1 - AUTHOR RESPONSE

Dr. Salvatore Paiella, University and Hospital Trust of Verona
Comments to the Author:
Thanks for assigning me to revise an interesting manuscript that deals with a topic I am keen on. The authors should be commended for the study and for having used the CWS in the context of pancreatic cancer surveillance, which is uncommon.

Please find below my considerations, comments, and concerns.

Thank you for taking time to review and comment on our manuscript. Please find our response below.

Minor:
1) The use of "individuals at risk", since the title, between quotation marks is unusual, whereas high-risk individuals is appropriate. The authors may be willing to use this locution throughout the whole manuscript. They use both acronyms, IAR and HRI, whereas the most adopted is HRI, and, anyway, only one should be chosen, preferably HRI.

Thank you for this comment we have adapted the throughout the manuscript accordingly.

2) Phrasing is sometimes poor, e.g.:
- There are two groups of IAR: familial pancreatic cancer (FPC) and hereditary cancer syndromes, with varying population prevalence and pancreatic cancer risk.
- However, individuals with a higher risk of developing cancer than the general population might
experience this differently (they should generalize and stay focused on PDAC surveillance)
- "to support these individuals better..." To better support these individuals.
- "Individuals participating in a screening program for high-risk PDAC, with no signs of active

tumor disease.": Individuals are at risk, not PDAC. What does it mean "with no signs of active tumor disease"?

- "They were contacted via the quality register, if still alive": of course, they were alive if they were contacted. Please rephrase or remove.

Thank you. We apologize for any poor language and have corrected this according to suggestion.

3) The paragraph starting with "Surveillance among" and closing with "pancreatitis attack" can be removed, and the reader will be addressed to current recommendations/guidelines. This will shorten the introduction, which is currently too long and would benefit from some cuts about general features of PDAC surveillance (modalities and inclusion criteria).

Thank you, for these suggestions we have amended the introduction for a better flow and a shorter version.

4) The authors state that "There is limited data on psychological impact related to screening for PC", referring only to the manuscript of Cazacu et al. They are missing other manuscripts published afterward: PMIDs: 38201514, 32108397.

Thank you for this suggestion the references have been added to the manuscript.

5) PDAC or PC?

We changed this and we are now only using PDAC.

6) The authors should state who selected the questionnaires to (self)administer. A psychologist? A psycho-oncologist?

The research group that conducted the study chose the questionnaires based on experience. This has been added in the text.

7) The phrase "In response, some programs – including the screening program in Stockholm – have started offering surveillance to individuals who do not meet the IAR requirement, but who express worry". What do the Authors mean? In a few countries, pancreatic cancer surveillance is considered standard clinical practice. In others, it is made within research programs, registered and IRB-approved. What is currently done in Sweden-Stockholm? The Authors may be willing to expand on this and state it clearly.

We agree with this comment and have clarified the purpose of the screening program in Stockholm accordingly.

8) Patient enrollment for prospective psychological evaluation: it is unclear if the Authors want to underline a selection bias or describe how patient selection was made.

We wanted to describe how the patient selection was made.

9) From "The participants were divided".. to "Cystic lesions". This part may benefit from a more concise and precise description of the risk categories of the HRI involved.

Thank you for this comment we have clarified the description of the categories in the text.

Main criticisms:
1) The main criticism is the inclusion of "the phenotype IPMN", which per sé is sibylline. What is a phenotype IPMN? MD-IPMN? BD-IPMN? Any info about worrisome features/high-risk stigmata? Ca 199 rise? Which are the genetic mutations the Authors refer to?
Is this population merging the reason why they chose "individuals at risk", underlying that a case-mixing was made including those having heredo-familial predisposition and cystic precursor lesions? The risk of malignant transformation is tremendously different regarding the various types of IPMN. The comparison of the psychological burden between this population and HRI with heredo-familial predisposition should be made prudently. Comparing the two main categories of HRI under surveillance sounds appropriate and scientifically valid, as well as among the categories of IPMN. Others have already made these comparisons.
Any comparisons and speculations among such different populations may be weak or clinically unsuitable, and the message delivered is misleading.

Thank you for your valuable feedback. We appreciate the opportunity to clarify our use of "phenotype IPMN" and the rationale for including this population in our study.

In our study, "phenotype IPMN" refers specifically to BD-IPMN (branch-duct IPMN) in individuals already classified as high-risk (HRI) due to hereditary or familial predisposition. These patients did not exhibit high-risk stigmata, elevated CA 19-9, or other malignancy indicators. Their inclusion was based on the premise that knowing they have pancreatic cysts might contribute to increased psychological burden, which we sought to investigate. No patients that were non-HRI but were included for surveillance for IPMN were included in this study.

Our comparisons were not meant to equate malignancy risk across groups but to explore the psychological impact of surveillance among individuals with different risk factors. To ensure clarity, we will explicitly state in the manuscript that only BD-IPMN without high-risk features was included.

We appreciate this feedback and will refine the manuscript accordingly.

2) The authors should correct their statistics for important variables that may ultimately impact the psychological assessment: a personal history of cancer (e.g., those HRI having genetic mutations may be more prone to have psychological distress), type of genetic mutation (HRD vs. Non-HRD), and family history of cancer (presumably rare in the cystic lesions group). Maybe the data are not large enough to achieve statistical significance, yet this deserves further exploration or at least mention among the weaknesses (which not mention at all the fact that "cystic lesions", in general, were included).

We completely agree that factors such as personal history of cancer, type of genetic mutation, and family history of cancer could have an impact on psychological burden, and we appreciate the opportunity to clarify these points.

Unfortunately, we did not collect data on personal or family history of cancers outside of PDAC, which means we are unable to assess how these might have influenced participants' levels of distress. We also recognize that different genetic mutations may contribute to varying psychological experiences, but this was not something we specifically analyzed in our study. While our dataset may not have been large enough to detect significant differences based on these variables, we acknowledge their potential relevance and will highlight this in our discussion.

To address your concerns, we will update our limitations section to explicitly state these points, ensuring that readers understand the scope of our analysis and the need for further research in this area. We truly appreciate your insights and believe these refinements will strengthen the manuscript.

Dr. Jenny Drott, Linköping University
Comments to the Author:
The paper investigates an interesting topic about the psychological burden in a pancreatic cancer surveillance program. Your research presents a significant contribution to the field of psychological burden in a pancreatic cancer surveillance program, offering valuable insights and advancing our clinical understanding of this specific topic.
The thoroughness of your methodology and the clarity of your results are particularly impressive, making this study a crucial reference for future research. Your work not only addresses key questions but also opens new avenues for exploration and supportive cancer care, underscoring its importance and relevance in women and younger individuals with more cancer worry and anxiety in a pancreatic cancer surveillance program.

Thank you for your time and your comments.

1. Why were patients not involved in the design of this study? How might their involvement have impacted the study outcomes? Consider adding in limitations.

The group of participants are not patients but individuals that have increased risk, we have had input on the design and inclusion of questionnaires from the patient organization PCE Pancreatic Cancer Europe and Swedish Palema for this project. This has been added to the manuscript.

2. How were the criteria for high-risk PDAC determined, and what evidence supports these criteria?

The criteria for high-risk PDAC are based on the CAPS 2020, referenced in the manuscript.

Goggins M, Overbeek KA, Brand R, Syngal S, Del Chiaro M, Bartsch DK, et al. Management of patients with increased risk for familial pancreatic cancer: updated recommendations from the International Cancer of the Pancreas Screening (CAPS) Consortium. Gut. 2020;69(1):7-17.

3. Consider clarifying the specific criteria used to classify participants into stringent IAR and non-stringent IAR subgroups.

Thank you for this comment, we have defined more clearly.

4. Consider clarifying how the risk of developing pancreatic cancer differ between the stringent and non-stringent groups, and the clinical implications of these differences.

Thank you for this comment. We usually say that Stringent HRI have a ≥5% lifetime risk or five-fold increased risk compared to the general population, meeting CAPS surveillance criteria, while Non-Stringent HRI have an elevated but lower risk, requiring individualized follow-up. Our recent data, upcoming publication, shows no difference in risk between Stringent and Non-Stringent IAR. We will also briefly discuss the clinical implications of these differences in the manuscript.

5. How was the quality control database at Karolinska University Hospital maintained, and what measures were taken to ensure data accuracy?

The quality control database at Karolinska University Hospital was maintained and updated by the two first authors, with updates finalized just before analysis. Data accuracy was ensured through systematic data entry and verification against electronic medical records.

6. What are the potential limitations of using electronic medical records to update participant information?

Not all relevant details may be consistently recorded, and documentation practices can vary between clinicians. There can also be delays in updates, and privacy restrictions might limit access to certain information.

7. You mention that your response rate in your study was high, with 67% of all those who were invited participating but also indicates that 33% of the patients did not provide data, it is potentially response bias? limit the generalizability of your findings?

In this type of questionnaire studies a 67% response rate is considered high and therefore the risk of response bias would be considered limited.

8. How were the questionnaires you used in the study validated for this specific population, and what are the potential limitations of using these instruments (in this specific population)?

The questionnaires used in the study has been used in similar populations in Sweden such as the CWS, the health questionnaires, SOC in other screening populations.

9. What are the ethical considerations of contacting participants via the quality register, and how was consent managed?

The study has received ethical permission, and the informed consent was signed electronically at the start of the questionnaire.

10. How might your results impact future pancreatic cancer screening programs?

Thank you for your question. Our findings highlight the psychological challenges faced by participants in pancreatic cancer screening programs. To enhance patient experience and ensure continued participation, it's crucial to integrate targeted psychological support into these programs. By addressing the specific needs of vulnerable groups, we can make screening more patient-centered and effective. We will elaborate on these implications in the discussion section of our manuscript.
